# Challenges for Circular Economy under the EU 2020/741 Wastewater Reuse Regulation

**DOI:** 10.1002/gch2.202200232

**Published:** 2023-05-26

**Authors:** Julio Berbel, Enrique Mesa‐Pérez, Pedro Simón

**Affiliations:** ^1^ Departamento de Economía Agraria Finanzas y Contabilidad Universidad de Córdoba, Córdoba Córdoba 14011 Spain; ^2^ Water, Environmental and Agricultural Resources Economics (WEARE) Universidad de Córdoba Córdoba 14011 Spain; ^3^ Departamento de Economía Financiera y Contabilidad Universidad Loyola Andalucía Dos Hermanas Sevilla 41704 Spain; ^4^ Director Técnico Entidad Regional de Saneamiento y Depuración de Murcia (ESAMUR) Complejo de Espinardo – Ctra. N‐301, Murcia Murcia 30100 Spain

**Keywords:** circular economy, regulation EU‐2020/741, water governance, water reuse

## Abstract

Wastewater reuse is seen as an opportunity to support a circular economy and optimize water resources. However, the use of wastewater is limited by the need for the proper protection of health and the environment and demands a certain minimum quality of treated reclaimed water. The objective of this work is to evaluate the opportunities both for the agents in the water treatment chain (from municipalities to farmers) and for technology providers under the recently approved Regulation EU‐2020/741. The new market and opportunities require new value chains, technology development, governance, risk assurance, and adapted local regulation. Bottlenecks also pose technological, environmental, institutional, economic, and social challenges. The identified needs and barriers must be properly addressed in order to accelerate the transformation of the water sector toward the circular economy. As a conclusion, Reg EU 2020/741 introduces minimum requirements for urban wastewater reuse and requires the definition of risk management and transparency. The real impact of regulation on circular‐economy objectives is limited by water scarcity and crop profitability. Social acceptance is critical for success.

## Graphical abstract

Reg EU 2020/741 introduces minimum requirements for urban wastewater reuse and requires definition of risk management and transparency. The real impact of regulation in circular economy objectives will be limited by water scarcity, downstream cascade reuse, and crop profitability. The social acceptance is critical for success.

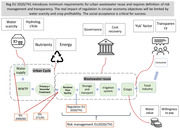

## 1 Introduction

Water scarcity is a growing concern worldwide due to the ever‐expanding demand for water and increasing water scarcity and pollution. Climate change and inadequate water governance increase the risk of water shortage. Nearly half the global population are already living in potentially water‐scarce areas.^[^ ^1^ ^]^ Water is also a limited resource in the EU.^[^ ^2^ ^]^

Globally, 80% of wastewater is discharged without treatment. The situation in the EU is precisely the opposite, where more than 80% of water is treated before discharge. However, part of this wastewater may undergo only partial treatment, and despite ≈20 million hectares are irrigated with untreated urban wastewater^[^ ^3^ ^]^ 200 million farmers use wastewater for irrigation.^[^ ^4^ ^]^ The need to address this global challenge is emphasized in Sustainable Development Goal number 6 of the 2030 Agenda.

Water reuse in general, and particularly urban reclaimed water reuse, may contribute toward reducing water stress when certain conditions are met. Energy, nutrients, and water can be recovered for their safe use in agriculture. Circular economy, or resource recovery, and reuse contributes toward a range of social, economic, and environmental benefits that affect human well‐being and is established as a critical strategy for meeting the goals of greenhouse gas reduction.^[^ ^5^ ^]^ The EU has recently approved a proposal for the Regulation 2020/741^[^ ^6^ ^]^ on water reuse for irrigation as part of the Circular Economy Action Plan.^[^ ^7^ ^]^

Non‐irrigation uses of reclaimed water include urban recycling (toilets, garden irrigation, etc.), public recreation areas, groundwater recharge, and direct and indirect potable use. Tortajada reviews the alternatives of water reuse and the link with SDG, while Duong & Saphores revise examples of direct potable reuse (purified wastewater is added directly to the potable water supply). Nevertheless, this document is focused on the review of reclaimed water reuse for agricultural irrigation and the likely impact of the new EU regulation on this activity. The main normative innovation discussed in this paper is the EU Regulation 2020/741 on Water Reuse that address exclusively reclaimed water reuse in agriculture and explicitly excludes other wastewater potential uses such as groundwater recharge or potable use.

The EU Commission Blueprint Report^[^ ^6^ ^]^ summarize the first decade of Water Framework Directive (WFD) implementation concluding that “alternative water supply options with low environmental impact need to be further relied upon” in order to address water scarcity.^[^ ^10^ ^]^ The Blueprint is considered the departing point of Regulation EU‐2020/741 and part of the Circular Economy Action Plan.^[^ ^7^ ^]^ This study aims to evaluate the opportunities and bottlenecks arisen with the publication of the Regulation EU‐2020/741 for wastewater agents involved in the water reuse process, that is, from municipalities wastewater plants till farmers and beyond (consumers).

Consequently, this study aims to evaluate the opportunities and bottlenecks arisen with the publication of the Regulation EU‐2020/741 for wastewater agents involved in the agricultural water reuse process (i.e., from municipalities wastewater plants till farmers).

The rest of the paper is structured as follow: next section reviews briefly the status of reclaimed water for irrigation meanwhile the third section analyses the role of reclaimed water in the circular economy. Section fourth describe the background and international examples of water reuse guidelines. In the fifth section there are exposed the critical issues that should face the EU Regulation. Sections sixth and seventh, conclude the work with the discussion and the concluding remarks.

## 2 Status of Reclaimed Water Use for Irrigation

Some countries (mainly developed economies) apply strict regulations regarding minimum requirements and safety standards, tolerating only high‐quality treated water to be used in crops, while the situation in less developed economies is less safe due to a lack of standards or to the inadequate enforcement and/or policy of the existing regulations. A recent review by Zhang & Shen highlights the long tradition of reclaimed water irrigation by quoting examples from 1700 BC, and also draws attention to the rapid development since the year 2000 fostered by public support, increasing water scarcity, and the technology available. ≈45% of urban reclaimed water is used for irrigation worldwide^[^ ^12^ ^]^ with the share of reclaimed water used for irrigation ranging from that in Israel (85%) and Spain (70%)^[^ ^13^ ^]^ to a much lower percentage (e.g., Florida with only 8%).

The positive effects of irrigation with reclaimed water include the creation of alternative resources where conventional resources are unavailable and the release of conventional sources when substituted by reclaimed water. Furthermore, crops enjoy an increased productivity due to the many nutrients available in the reclaimed water and this helps farmers (and therefore the environment) since it obviates the need to supply nutrients that usually imply fossil resource consumption and the generation of greenhouse gases. The negative effects include the possible contamination of the soil (salts, heavy metals), possible lixiviation of nutrients when irrigation is applied inadequately or in excess. Negative effects can be prevented by using efficient irrigation systems such as drip irrigation (standard or underground) and by controlling the water supply to halt over‐irrigation and over‐fertilization, which are responsible for diffuse pollution from irrigated plots and can be prevented by rational agronomic practices. Finally, health risks (both human and environmental) can be mitigated with the use of suitable treatment.

According to Zhang & Shen, the challenges facing the practice of irrigation with reclaimed water include: a) the provision of appropriate technologies for reclamation facilities; b) adaptation to climate change; and c) the introduction of suitable and stricter laws and regulations on reclaimed water irrigation. Beyond technical measures (a) and (b), the improved governance required by (c) implies the participation of stakeholders across sectors (urban industry, farmers, food industry, consumers) on various geographical scales (local, regional, national) and the inclusion of different perspectives (consumers, administration, producers, and the environment). The lack of public support the cause of the failure of several water reclamation process in USA or Australia.^[^ ^14^ ^]^ Additionally, certain institutional changes are required regarding the definition of: property rights (who owns the water), the priority of users (seniority rules in case of drought events), cost recovery, and the role of responsibility for quality assurance (reclaimed water requirements).

### 2.1 Reclaimed Water Uses for Irrigation in the EU and Other Countries with a Mediterranean Climate

According to a recent report,^[^ ^15^ ^]^ the WFD has improved the status of water in the EU and has been especially successful in reducing point source pollution since 88% of EU urban wastewater is subject to secondary treatment. However, water reuse remains low in the EU,^[^ ^15^ ^]^ estimated as being low (2.4%) compared to other countries such as China (14%), the US (14%), Australia (15%), Singapore (35%), and Israel (87%) (data taken from various sources). This low use of reclaimed water in the EU may be explained by the fact that most EU countries are water abundant and resource scarcity is largely concentrated in Mediterranean regions.

Reclaimed wastewater is used in regions that do not classify as Mediterranean climate, that is the main focus of this article, a recent and complete review can be found in^[^ ^16^ ^]^ with an comparative analysis of standards in Asian, Middle east, and American countries that also have legislated on the water reuse quality for agriculture., beyond agricultural use, other sectors also reuse water, mainly industrial cooling applications.

Around the Southern EU ≈20% of the population lives under water stress, and, by 2030, water stress and scarcity will probably affect half of Europe's River basins.^[^ ^10^ ^]^ Reclaimed water use is seen as an instrument that may alleviate scarcity in certain regions.^[^ ^17^ ^]^ Over 16 of the EU Member States for which data are available (EUROSTAT), more than 80% of the EU population is connected to at least secondary wastewater treatment plants, which typically grants an acceptable level of environmental protection unless the receiving waters are in a sensitive area. More than 40 000 million m^3^ of wastewater is treated in the EU every year but only 964 million m^3^ (2.4%) of this treated water is reused.^[^ ^18^ ^]^

Cyprus and Malta already reuse more than 90% and 60% of their wastewater, respectively; other non‐EU Mediterranean countries, such as Israel, recycle almost 100% of their wastewater. Larger EU countries, such as Greece, Italy, and Spain, currently reuse between 5% and 12% of their effluents, which indicates an opportunity for greater reuse. An assessment of potential water reuse for agriculture in the EU, which draws an optimistic scenario for reclaimed water cost below 0.5 € m^−3^ (investment, operation, and transport, excluding storage), shows an expected use of 6.6 hm^3^ for the EU, whose distribution is shown in **Table** **1**.

**Table 1:** Potential WW reuse in the EU

% WW reuse / total treated WW	Volume WW [hm^3^]	reuse if cost <0.5€/m^3^ [hm^3^]	% WW reuse	Countries
> 20%	19 319	5714	29.6%	MT, PT, IT, ES, GR
[10%–20%]	5190	608	11.7%	SK, FR
[5%–10%]	1570	105	6.7%	DK, NL
[1%–5%]	8750	154	1.8%	BE, HU, AT, DE
[<1%]	13 808	36	0.3%	Rest of EU
Total EU	48 640	6619	100%	

Source: Adapted from (Pistocchi 2017).

The scenario of wastewater reuse, as summarized in Table 1, has provided the basis for the socio‐economic impact assessment of the Regulation. The computation of water demand for irrigation considers the irrigation demand available up to the cost threshold. The scenario assumes that 5.5 hm^3^ (80% of the expected EU reclaimed water) will be used in Italy (IT), Spain (ES), and Portugal (PT), and considers the substitution of current sources of water (mainly groundwater) with reclaimed water but does not explain how any farmer would be willing to accept a change of water supply from the current cost that is in the range 0.02 to 0.10 € m^−3^.^[^ ^19^ ^]^ In certain locations, reclaimed water will probably be employed to expand irrigation, and, in the case where no other alternative is available, users will probably consume reclaimed water. This point is further is the core of the next section. The use of non‐conventional water (desalinated and reclaimed) increases the consumption of energy as consequential to the water‐energy nexus.^[^ ^20^ ^]^ The advantages of using reclaimed water for agriculture has been demonstrated in regions with Mediterranean climate such as Australia, where the Millennium drought catalyzes the reuse of water proving to be effective both for reducing nutrient loading to the Melbourne bay and for augmenting the water supply for the city.^[^ ^21^ ^]^

## 3 Circular Economy and Wastewater Resource Recovery

The Circular Economy (CE) aims to maintain the value of resources during the whole the product lifecycle and to minimize the generation of waste. Regarding the application of CE principles to the water sector, the preferred option for the EU regarding water scarcity and droughts is to control and reduce demand, but a combined approach, that additionally includes measures to optimize existing resources within the water cycle and measures to create new resources (water reuse, desalinization), is also admissible.^[^ ^22^ ^]^

### 3.1 Implementing CE in the Water Sector

Water is essential to the CE due to the energy and material that it contains and because water scarcity is increasing worldwide. Water scarcity affects 11% of the population of the EU and 17% of its territory.^[^ ^10^ ^]^ The EU has declared the importance of water and wastewater management as a priority for the CE.^[^ ^23^ ^]^ The original focus of the EU was both on resource‐use efficiency (water saving) and on the recovery of raw materials and energy in wastewater. Regarding water scarcity objectives, maximization of water reuse is a specific objective in the Communication “Blueprint,”^[^ ^6^ ^]^

As will be seen later, there are both “hard” (capital intensive) and “soft” (direct application of sludge, composting) technologies available to maximize the capture of nutrients and energy. In addition to the technological options, there is, however, a need for institutional and social involvement to make the implementation of CE principles successful. Fulgenzi et al. reviews the characteristics of stakeholder involvement through the Community of Practice (CoP) so that governance promotes the participation of stakeholders from relevant sectors and territorial levels who contribute different viewpoints and preferences into the governance system. The available literature indicates that further research into governance systems is required for the design of sustainable and equitable structures.^[^ ^25^ ^]^

Water reuse requires a clear and proactive definition of water rights.^[^ ^26^ ^]^ Allocation regimes determine who is able to use water resources, how, when and where,^[^ ^27^ ^]^ this includes explicit consideration of characteristics such as priority for water use in drought, which is frequent in Mediterranean regions and is becoming increasingly frequent in the EU.^[^ ^28^ ^]^ The fact that reused water is less affected by droughts and fluctuations in water availability remains an incentive to obtain water entitlements. However, since reused water is usually more expensive than conventional sources (surface, groundwater), it tends to be neglected in years of abundance.^[^ ^29^ ^]^ We review the role of allocation later in the section focused on governance.

The CE in the water sector goes beyond the inclusion of additional quantitative water resources, and it has at least four different dimensions: a) nutrient recovery; b) energy; c) water resources; (quantitative), and d) sewage sludge valorization. The implementation of the CE in these areas is compatible and synergistic with the maximization of reclaimed water reuse for irrigation. The opportunities for CE in these areas are described below.

### 3.2 Nutrients and Other Material Recovery

Wastewater is rich in nutrients such as nitrogen, phosphorus, and potassium that can be a resource as a fertilizer required by plants, but it can also become a serious environmental problem when released in the environment without proper treatment. ≈47% of (semi‐)natural ecosystem areas were subject to nutrient nitrogen deposition leading to eutrophication in 2004.^[^ ^30^ ^]^ In many of the EU regions countries, phosphates and nitrates are the main pollutants in water bodies.^[^ ^31^ ^]^ Generally, agriculture is the main responsible through nitrogen diffuse pollution (as phosphorus is retained by agricultural soils). The impact of urban wastewater mainly in phosphorus but also nitrogen pollution varies according each watershed characteristics, and it is recognized that in some regions, urban and industrial wastewater maybe be responsible for the majority of phosphorous reaching water masses.^[^ ^32^, ^33^ ^]^ We can avoid pollution downstream and maximize the recovery of materials, by capturing both nutrients and other materials in the source (WTTP).

Regarding material recovery from wastewater, phosphorus is included as a critical raw material the emphasis is done in most critical material as it is required for food production and the EU is dependent on outside providers some of them with unstable policy regimes^[^ ^23^, ^34^ ^]^ and it can thus be expected that recovery from wastewater will gain importance.

Nutrient recovery management under a CE framework can be seen as a cycle comprising the following stages: 1) production (e.g., Nitrogen fertilizer); 2) agricultural use; 3) processing; 4) consumption; 5) treatment of waste and wastewater; 6) return of nutrients to agriculture. The most widely used method for the capture of nutrients is the formation of struvite (magnesium ammonium phosphate), this is done frequently with the addition of elements such as magnesium. Other reagents less frequently used are such as calcium, aluminium, and iron. An exhaustive and review can be found in.^[^ ^35^ ^]^

Once nutrients are recovered, operators need to deal with a fragmented a complex regulation and authorization processes for the recovery plant and the marketed nutrients. Hukari et al. review the EU legislation governing phosphorus recycling that can be also applied to other nutrients in the wastewater. Fertilizers produced from waste meeting quality, safety and other standards can be traded freely within the EU.^[^ ^15^ ^]^ Some food and pharmaceutical industries wastewater can also have high value products, but the Regulation EU 2020/751 regulates only wastewater from urban treatment plants.

The use of reclaimed water is a cost‐efficient alternative for re‐integrating nutrients such as N, P, and K required by plants. Reusing water in the terrestrial environment (e.g., irrigation) helps reduce environmental degradation of lakes, rivers, streams, and coastal waters. Plants can use nutrients contained in the reclaimed water, which can reduce the amount of nutrients directly discharged to natural systems and simultaneously can lead to a reduction in nitrogen and phosphorous fertilizer applications, resulting in lower costs at farm and global levels and lower environmental impact. Farmers should take into account the supply of N, P, and K received from reclaimed water, and these nutrients should be counted as part of the overall fertilizer program to avoid “overfertilization.”

### 3.3 Energy

In the EU‐27, 69% of the population were connected to tertiary level treatment and 13% to secondary level treatment (adding to 82% connected to a WWTP).^[^ ^30^ ^]^ This level is quite similar to USA where ≈80% of population receives sewage treatment services from municipal WWTPs contributing to more than ≈4% of the entire US electrical demand.^[^ ^37^ ^]^ Electricity demand for WWTP was about the 0.8% of the electricity consumption in the EU‐28.^[^ ^38^ ^]^ A recent worldwide survey of energy use in WWTP conclude that “(some) EU states had the largest average (water consumption) kWh m^−3^ with 1.18 which appeared a result of the higher wastewater effluent standards of the region. This was supported by Denmark being the second largest average consuming country (1.35 kWh m^−3^), since it has some of strictest effluent standards in the world.”^[^ ^39^ ^]^

According to some estimates,^[^ ^40^ ^]^ worldwide ≈65 WWTP have reached zero energy emissions, although this is encouraging, still a minority of WWTP in both the US and Europe achieve energy self‐sufficiency.^[^ ^41^ ^]^ Assuming that the average energy consumption in WWTPs is 0.85 kWh m^−3^, the water can contain up to 12 times more energy than what is needed for its treatment.^[^ ^35^ ^]^ Consequently, better management of WWTP including optimization of energy use (pumps, aerators) coupled with a better management of biogas generation may be able to achieve 100% energy self‐sufficiency in most of the WWTP in medium and large cities.

The use of targets for environmental policy is common in other EU policy fields. In December 2019, the European Commission presented European Green Deal, committing to climate neutrality by 2050. The Urban Wastewater Treatment Directive^[^ ^42^ ^]^ supposed the improve of rivers, lakes and seas quality. The proposal for revising the rules on treating urban wastewater aims (among other considerations related to human health and environmental protection) to make the wastewater sector energy‐neutral and move it toward climate neutrality.^[^ ^42^ ^]^ This is a link between water and energy efficiency but some critical decision should be made in the field of neutrality in WWTO as Li et al. argue that the definition of system boundary that may go from a narrow analysis focused strictly “Within‐the‐fence of WWTPs,” a wider analysis that expand the energy analysis to urban infrastructure related to WWTPs or a more global approach expanding to human society and ecological system, the second and latest boundaries may include wastewater reuse including carbon capture by crops and energy savings from nutrients recycling by crops.

To conclude, the future of WWTP will be a search of self‐sufficiency in energy use that is compatible with the goal of increasing nutrient recovery and water reuse. The maximization of energy generation In WWTP is compatible and synergic with the use of reclaimed water in agriculture.

### 3.4 Water Resources

A critical distinction the concept of indirect and direct reuse of treated wastewater, also called as unplanned or planned reuse, respectively.^[^ ^44^ ^]^ As mentioned above, reclaimed water should be considered in the global context of hydrological cycle and basin management. In practice, from the point of view of new resources in water scarce region, only WWTP close to the final sink (generally the sea) can be used for water reclamation and the water can be considered an “additional resource” for the basin.

According EUROSTAT in year 2011, 41% of EU population lived in a coastal region (NUTS3) and 35% is living close to the sea (>50 km) which can be used as an upper range estimator of the population where reclaimed water maybe consider as an additional resource, with inland regions and cities as location where return flows from WWTP should not be considered a new resource as it is already accounted in the hydrological cycle. The volume of potential reused water when considering this 35% of population should also consider the real demand that is concentrated in Mediterranean and water scarce regions, we should consider that irrigation in Scandinavian and Northern EU is scarce and very limited to some drought events.

There is a high risk if not considering basin view and the indirect or planned water reuse that we have mentioned. It is of great concern the lack of basin view when estimation of water reuse is produced by public or private bodies. The important publication by JRC^[^ ^45^ ^]^ neglect any mention to existing indirect reuse of wastewater under current Dir 91/271. Water Agencies in stressed basins such as Guadalquivir (Southern Spain) do not allow any additional direct reuse of wastewater as the urban treated return flows are already required for maintaining environmental flow and are included in downstream water resources.^[^ ^46^ ^]^

The cascading water use in river basins is one of the main constraints for the setting and practical implementation of water efficiency targets at the basin level.^[^ ^47^ ^]^ Usually, in river basins, the return flows from upstream water users to the river (or lake or groundwater body, for example, due to water infiltration) are used as an input resource for downstream users. In consequence, a river basin system often has a different water use efficiency than its individual components, and low efficiencies in upstream areas ensure water flows in downstream water bodies. As current use of the non‐consumed fraction of applied irrigation water and the ecosystems and economic uses of the return waste stream.^[^ ^48^ ^]^ The integration of wastewater reuse in basin management has been treated scarcely in the literature, with just a few exceptions such as.^[^ ^49^ ^]^

In case that indirect reuse is already accounted in the basin water balance new direct water reuse will negatively impact the river flows and does not be considered ‘new non‐conventional resources. This common disregard of basin view and hydrological cycle is found in a great share of circular economy analysis of water reuse such as the mentioned original JRC report^[^ ^45^ ^]^ and the related recent report by.^[^ ^50^ ^]^ Unfortunately academic literature frequently neglect this critical issue with no mention of water balance and basin reuse neither in a recent review on the topic^[^ ^16^ ^]^ the term is mentioned, this may be partially justified (although it should have been addressed) in water abundant regions such as northern Italy^[^ ^51^ ^]^ but is a serious shortcoming in water scarce regions where environmental flow is already a constraint in the water use and any additional abstraction or reduced return flows increased water shortages such as^[^ ^52^ ^]^ in central Spain or^[^ ^53^ ^]^ in Korea.

Against to this omission from academic and technical domains, water agencies in stressed areas take seriously into consideration the indirect reuse in the basin water balance. Some examples of Water Agencies management of the wastewater reuse in stressed basins are Guadalquivir (Southern Spain) where is not allowed any additional direct reuse of wastewater as the urban treated return flows are already required for maintaining environmental flow and are included in downstream water resources.^[^ ^46^ ^]^ Another example maybe Nevada (USA) where water rights are defined as water withdrawals (or diversions) minus any water that is returned to the Colorado River. These returns are also known as “return‐flow credits” and urban wastewater is included in the ‘credits’ that in case of “direct reuse” the volume is deduced form the user balance.^[^ ^54^ ^]^

Additionally, many analysis of direct wastewater reuse include the “water footprint concept” without any reference to basin water balance falling in an common mistake when using this problematic concept that is originated in economic literature but that has been criticized from the hydrology science^[^ ^55^ ^]^ and agronomic science.^[^ ^56^ ^]^

### 3.5 Sewage Sludge

Sewage sludge, also known as biosolids generally have lower nutrient contents than commercial fertilizers. Biosolids typically contain N (3.2%), P (2.3%), and K (0.3%).^[^ ^57^ ^]^ In the EU, according to EEA,^[^ ^30^ ^]^ the main sewage sludge treatment method is the direct application for agriculture (Ireland, 79 %), composting (Estonia, 84 %), incineration (Netherlands, 98 %), or landfill (Malta, 100 %), in the USA, land application is 41%, composting 20% and disposal (landfill, incineration) is 40%.

The amount of sewage sludge that can be used directly in agriculture or indirectly as fertilizers is subject to limits provided in the EU and national regulations (see section 3.1 above). Specifically, in the EU the Sewage Sludge Directive 86/278/EEC encourage the use of sewage sludge in agriculture and regulates the safe application. In 2014, the Directive was evaluated as part of an “Ex‐post evaluation of certain waste stream directives.”^[^ ^58^ ^]^ The report shows that biosolids use in agriculture has been positive regarding nutrient recycling and the heavy metals have not exceed the limits set by the Directive. The EU has plan for 2021 a revision to improve future integration of this waste in the Circular Economy. The recycling of biosolids is compatible and synergic with the use of reclaimed water in agriculture.

### 3.6 Bioeconomy

Microalgae cultivation has been proposed for nutrient removal in municipal wastewater treatment^[^ ^59^, ^60^, ^61^ ^]^ and they have been also evaluated for removal of pharmaceuticals from wastewater.^[^ ^62^ ^]^

Additionally, the use of microalgae can go beyond nutrient removal as wastewater may reduce the cost of microalgae production allowing bioeconomy products such as biofuels, biofertilizers, or bioproducts. The use of microalgae in urban wastewater treatment is a promising field that is currently is focused in the development of robust, productive wastewater‐adapted microalgal species, and the engineering for cultivation and processing systems that optimize harvesting and conversion of the algal biomass.^[^ ^63^ ^]^ The conversion of WWTP to “bio‐factories” has been announced in locations such as Santiago de Chile (Suez company) or the “All‐gas project” (Aqualia company, Spain) just to quote some real‐world examples.

## 4 Foundation of the EU Regulation on Reclaimed Water Use

### 4.1 Background and Other International Examples of Water Reuse Guidelines

**Figure 1:**
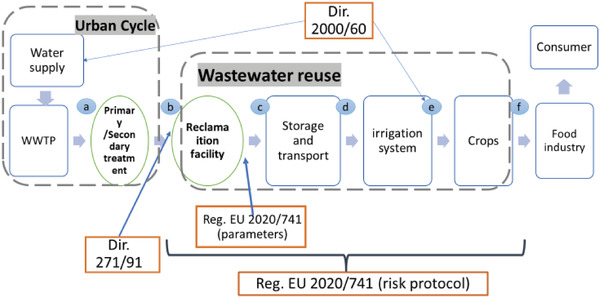
Main elements of Reg. EU 2020/741 wastewater reuse owns elaboration.

Reclaimed water use has been regulated by state agencies and supranational institutions dating back to the 1970s. The main features of the Reg. EU 2000/271 are illustrated in **Figure** **1**, where the regulation is related to the closely linked Dir 271/91 and Dir 200/60.

An initial attempt to regulate the use of wastewater was carried out by WHO in 1978 with a definition of very strict guidelines that were later transformed in the more practical “Health Guidelines for the Use of Wastewater in Agriculture and Aquaculture”^[^ ^64^ ^]^ aimed toward providing safe reuse in developing economies. The standards were adopted later by FAO in a report “Wastewater Treatment and Use in Agriculture” published in 1992.^[^ ^65^ ^]^

In addition to these supranational institutions, the state of California is a pioneer regarding water reuse due to its regulations held since 1918 as the Californian standards, namely California Code of Regulations Title 22. These regulations constitute a milestone of required treatment of various types of reuses to ensure a pathogen‐free effluent in the form of a combination of biological wastewater treatment followed by tertiary treatment including filtration. This California code serves as a benchmark since it sets the highest standards on the quality of drinking water and was influential in the formulation of the^[^ ^66^ ^]^ for reuse.

California standards were adopted by other countries, such as Spain, whose national law provides regulations for the reuse of water^[^ ^67^ ^]^ and follows California regulation although with less strict requirements. Other countries in the EU have regulated reclaimed water use for irrigation. **Table** **2** gives a summary of existing regulations and the critical parameters for Class A for all food crops consumed raw where the edible part is in direct contact with reclaimed water, and for root crops consumed raw and applied to all irrigation methods.

**Table 2:** Total requirement for Class A water reuse

Parameter	EU 202/741	CY	IT	PT
E coli cfu/100 mL)	< 10	< 1–10	<10	–
Faecal coliform	–	<50	–	<100
TSS [mg L^−1^]	<10	15‐30	–	<60
Turbidity (NTU)	<5	–	–	–
BOD5 [mg L^−1^]	<10	–	<20	–
COD [mg L^−1^]	–	<60	<100	–
Total Nitrogen [mg L^−1^]	–	<15	–	–

Source: EU 2020/741 and national regulations.

The EU Reg. 2020/271 tries to capture previous international and national regulations in wastewater reuse (e.g., WHO, FAO, Spanish or Italian regulations) and incorporates the existing parameters regarding nutrients and pollutants, but as we will see below, goes beyond parameters threshold (see Table 2) and incorporates a novel system of risk assurance protocols that is essential part of the EU normative. The combination of these requests makes the EU 2020/741 one of the most demanding norms in this field worldwide. A recent report^[^ ^68^ ^]^ revising the legislative framework in EU regions detects that minor changes are required in most of the EU Southern States regarding a) permits and competent authorities, b) classes and requirements for reclaimed water, and c) monitoring requirements but major changes are required in d) validation of monitoring, e) Risk management plan, and f) information to the public.

### 4.2 The EU Regulation on Wastewater Reuse

The EU regulatory powers for internal markets are exercises based on various normative instruments and generally approve ≈80 directives and 1200 regulations per year. Directives are the most important and most general and need to be subsequently transposed into national law, while regulations may range from handling trivial administrative matters to regulating the market development of major sectors. This is the case of the Regulation on wastewater reuse, which critically influences the development of this sector both within and outside the European Union.

In the water sector, there are several critical directives, such as the Water Framework Directive (2000/60/EC), Urban Wastewater Directive (91/271/EEC), Drinking Water Directive (98/271/EEC) Groundwater Directive (32 006/118/EC), Bathing Water Directive (2006/7/EC), Industrial Emission Directive (2010/75/EU), and the Marine Strategy Framework Directive (2008/56/EC), which should be borne in mind since they are all closely related to the WWR regulation.

For directives to be applicable, they need to be transposed into national law. Regulations, however, are binding legislative acts from the beginning, and must be applied in their entirety across the EU, with no transposition necessary. For example, when the EU wanted to make sure that there were common safeguards on goods imported from outside the EU, the Council adopted a regulation, and it was applied at all EU borders immediately. This is the case for the WWR regulation, whose objectives are defined below. The EU has decided to address the wastewater issue as “regulation instrument” that implies uniform implementation for all Member States avoiding the complex process of Directive transposition where some local differences may appear, nevertheless EU regulation still requires national and regional incorporation of principles, standards, and risk guidelines to be incorporated in operating rules, the licensing of facilities, and operator training procedures that require national development.

According the EU, “the purpose of this Regulation is to guarantee that reclaimed water is safe for agricultural irrigation, ensuring a high level of protection of the environment and of human and animal health, promoting the circular economy, supporting adaptation to climate change, and contributing to addressing water scarcity and the resulting pressure on water resources, in a coordinated way throughout the Union, thus also contributing to the efficient functioning of the internal market.”^[^ ^6^ ^]^

In the paragraph above, the main elements that support the existence of a common regulation can be observed:

- a high level of protection
- promotion of the circular economy
- support of adaptation to climate change
- contribution toward addressing water scarcity
- contribution toward the efficient functioning of the internal market

It can be observed that a single instrument aims to achieve or contribute toward a variety of objectives, and this paper strives to evaluate the level of conflict, complementarity, and role of this regulation in promoting WWR in the EU. This paper aims to analyze the state of the art of WWR in the EU, and the expected barriers and opportunities that the EU regulation introduces.

The European Commission has proposed technical guidelines for the application of the key risk management principles linked to a water reuse system,^[^ ^50^ ^]^ this topic is developed later in an specific section.

## 5 Critical Aspects of Regulation EU‐2020/741

### 5.1 Public Participation in the Regulation and Critical Aspects

The procedure to approve this regulation has undergone extensive public consultation, with several of the main stakeholders arguing the role of critical barriers: farmers expressing their worries concerning consumer perception and legal liabilities; irrigators proposing that the regulation also be applied to other users (industry, services); water utilities focusing on administrative issues (permit systems, as utilities claim that they are not interested in irrigation and should therefore not be held responsible for this administrative process); and the distribution of responsibilities regarding risk management plans. These are some of the questions raised during the stakeholder consultation process.

Mesa‐Pérez & Berbel^[^ ^69^ ^]^ present an analysis of public perception and find heterogeneous views linked to the regional system conditions. Based on a SWOT analysis combined with cluster of perceptions and given the abundance of water and agriculture competitiveness, the global perception is that a) the high cost of reclaimed water for farmers, and b) social fear of accepting products irrigated with reclaimed water should constitute the keystone of the EU strategy to foster the use of reclaimed water in agriculture. The analysis of published research on stakeholders’ perceptions leads us to propose a few dimensions that may be critical for the success of WW reuse in the EU. These are given in greater detail below and focus on four dimensions: 1) public perception; 2) water governance; 3) risk management; and 4) financing and cost recovery.

### 5.2 Public Perception

The approach to water reuse is in danger of focusing on technological solutions and problems that minimize the importance of social issues. This approach can be a serious mistake. The risk of consumer refusal of food irrigated with reclaimed water has been mentioned as a critical factor in the process of the Regulation design^[^ ^14^ ^]^ and has also been a frequent concern of farmers and those involved in the agri‐food chain, as mentioned by stakeholders in the consultation process.

An extensive review of consumer attitudes was conducted by CSIRO for Victoria State.^[^ ^70^ ^]^ They found that those people with a positive and active attitude toward environmental concerns and conservation were more likely to be accepting reclaimed water. However, a distinction should be made between reclaimed water for direct use, when water is used directly (e.g., public drink or aquifer recharge for potable use), and for indirect uses, when it is discharged on another water mass (like a river) before using it (e.g., irrigation).

Direct uses are usually associated to a factor known as “yuk factor” that reduce the positive attitude toward reclaimed water when the uses proposed are cooking or drinking. The evidence of failed projects demonstrates that higher levels of trust in the water authority are associated with lower perceptions of risk.^[^ ^71^ ^]^ The reason can be found on the risk perception. Research on consumer attitudes found that “Consumers prefer not to know” as their willingness to pay is greatest when the wine is made from grapes irrigated with an unspecified type of water.^[^ ^72^ ^]^ A similar result is found for fresh produce.^[^ ^73^ ^]^ Mainali et al. analyzed the factors of success and failure of several project of reclaimed water implementation. This concludes that the lack of information and education is the main factor that suppose the failure.

Indirect reuse is more easily accepted as Li et al. found that consumer perceptions of riskiness varies regionally and that the use of non‐traditional water diminishes consumer demand by 87% in the United States and by 20% in Israel for food irrigated with non‐conventional sources (reclaimed and desalinized water). Analysing the factors of success,^[^ ^74^ ^]^ found out that they promote a legitimation approach focusing on technology and social marketing, with the aim of increase “public acceptance.” Potable reuse is supported by the public when a transparency and accountability are guaranteed as evidence in Groundwater Replenishment System shows in California system.^[^ ^71^, ^74^ ^]^

The terminology employed to label reclaimed water may influence its perceived risk, with recycled preferred to reclaimed water in a large USA panel.^[^ ^75^ ^]^ Certain results suggest that the common names for treated wastewater, such as Recycled, Reclaimed, and Treated wastewater raise the level of rejection, while names that invoke desirable characteristics (Pure, Eco‐Friendly, and Advanced Purified) are preferred by the consumer.^[^ ^76^ ^]^ This result is consistent with the findings by^[^ ^77^ ^]^ who detect that willingness to use was reliably higher with the recycled water descriptor for both farmers and consumers.

To summarize this section, it can be concluded that: a) terminology is important; b) consumers prefer to remain unaware of the origin of the irrigation water since there is general disgust for food grown with reclaimed water; and c) public trust in authorities reduces the perception of risk. These conclusions are significant since recycled water is frequently used for irrigation mixed with other sources (surface water, groundwater, desalinated water) and the labeling of crops irrigated with reclaimed water can be difficult in practical terms and may even act as a barrier to use. The same occurs when recycled water is used for managed aquifer recharge that later serves to irrigate crops. This issue regarding third‐world countries with lower requirements will be raised in the future as a demand for fair competition from the farmers.

Another relevant point is the perception by consumers of economic circularity principles when consuming any product and service. The recycling of nutrients (N, P, others) in the use of reclaimed water deserves a mention as an example of the circular economy that is often valued by the consumer as a positive characteristic of reclaimed water use. Finally, a policy of transparency and openness to the public is critical to overcoming the reluctance of consumers.

### 5.3 Water Governance

Water governance refers to the political, social, economic, and administrative systems in place that influence the use and management of water. This includes who attains what water, when and how, and who has the right to water and related services, and their benefits and how to solve conflicts in the case they arise. Regarding reclaimed water, governance includes a clear definition of legislation and institutions, precision in the responsibilities of government, the private sector, and society in relation with water services, risk prevention, and conflict resolution. The implication of several bodies and institutions implies a coordination effort, moreover, when, as seen in the subsection 5.2, it is needed a coordination and transparency of the processes to gain public acceptance. Consequently, to achieve the challenges we are going to expose in this section, it could be useful the implementation of adaptative governance practices. In other words, public participation based on public administrations and stakeholders’ collaboration with the aim of learn and reach the issues^[^ ^78^ ^]^ in this case, of fostering reclaimed water use.

A critical decision involves the definition of property rights regarding water quantity and responsibilities in the maintenance of quality. Deficient treatment of wastewater may suppose a risk to farmers, consumers, and to the environment. Consequently, the risk management protocol should clarify rights and obligations, the price of services, and the cost of failure in meeting standards. Additionally, risk assessment can go beyond the reclamation facility since even the best water in the world can become polluted if improperly managed (e.g., mixed with livestock discharge). This aspect is a critical point in the implementation of the EU 2020/741 regulation.

Economic development has exerted growing pressure on water resources which has driven many river basins and aquifers to a state of closure,^[^ ^79^ ^]^ thereby increasing the need for effective management of the limited resources. Easy “politically correct” solutions may not work since local water‐saving measures fail to function without an understanding of the impacts downstream, as the debate around the rebound effect of water conservation investment illustrates.^[^ ^80^ ^]^ The reuse of water from a WWTP should consider the point of discharge, the existence of water reuse by downstream agents, and the impact of changes in the resource destination on the environment.

The key question regarding the allocation of water rights involves the definition of the owner of the return flows (wastewater discharge). It is generally assumed that most water abstracted for the urban cycle is discharged (over 80% is often assumed). These return flows, when properly treated, may be allocated to alternative uses, such as economic uses, the environment (environmental flow), and a new demand. When reclaimed water is used in irrigation, a large percentage of the water used is consumed through evapotranspiration depending on the level of deficit irrigation and system efficiency.^[^ ^80^ ^]^

When reclaimed water is allocated to a new irrigated area or to the increased supply in a previously deficit system, the global consumption from the basin is increased which can be acceptable either in the case of a water‐abundant system where environmental flow is not affected or in the case that the return flow is discharged into the sea or a similar destination (salt‐water masses). Therefore, new demand served by reclaimed water is not in conflict with previous water rights (economic users) or environmental quality when the discharge is either in the sea or in water‐abundant regions.

The problem arises in inland water‐scarce areas (not close to the sea) when all resources are already allocated, and new demand can only be satisfied by reducing allocation to previous users (either economic or environment). This is the context known as “closed basins” which is frequent in the Mediterranean and water‐stressed regions. The allocation of reclaimed water to new users in this context may increase basin consumption and constitutes a case of rebound similar to that described in the literature for water‐saving technologies.^[^ ^55^, ^80^ ^]^ The critical point here is to integrate reclaimed water into the hydrological cycle; this is carried out in Spain in all hydrological plans.

Many initiatives already consider this situation and aim to substitute existing the water supply from over‐allocated or over‐exploited sources, frequently groundwater. In this context, the policy is directed toward changing water from the over‐abstracted aquifer, but the problem arises when the final cost of reclaimed water (treatment, storage, and transport) is significantly higher than that of the previously existing sources. One solution involves the integration of wastewater in a mix of resources (surface, groundwater, and eventually desalinated) so that water supply and demand is managed in an integrated way. This requires complex management that is difficult to implement but that has been successfully carried out in certain locations. The successful implementation of this integrated system has resulted in a reduced cost and improved sustainability.^[^ ^79^ ^]^

Part of the solutions may involve using a managed aquifer recharge (MAR) to distribute the water for farmers instead of using a large distribution network. In this solution, the problem is the mechanism of cost recovery that finances the scheme. Cost recovery can range from a full‐cost pricing scheme, such as that found in Israel, or by subsidizing the service, as in Cyprus.^[^ ^81^ ^]^ The economic policy is analyzed later in this section.

To summarize this section, the policy decision regarding governance should define the water allocation rules by considering the full hydrological cycle and the mechanism for cost recovery and management of alternative and integrated sources. One critical issue involves the current position in several member states (e.g., Spain) regarding the compensation of water rights from reclaimed water by an exchange with existing rights, (e.g., groundwater). This makes it very difficult to increase reclaimed water use. A flexible and efficient system is critical for water right entitlement under the River Basin Authority. It is necessary to promote the rights exchange from fresh water to reclaimed water or allow the aquifer recharge including a future right of that water. Reclaimed water provides more opportunities to fight water scarcity, but it also requires the modernization of water rights.

The Urban Wastewater Directive 271/91 defines a level of discharge limits that is much more tolerant than the EU‐2020/741. Indeed, it is cheaper to discharge into a river or sea under Dir. 271/91 than the additional treatment required for it to be used as reclaimed water. The approval of a stricter regulation for Wastewater discharge (271/91) will transfer part of the reclamation cost to the urban user and will also substantially improve the quality of water masses in the EU. This will also improve quality of surface water that is usually mixed with reclaimed water with the paradox that reclaimed water with substantially higher quality than surface water when the urban and industry discharges in the river remain under the levels stated in Dir. 271/91.

The issue of quality control and risk monitoring also forms part of the governance; this is addressed in greater depth in the section below.

### 5.4 Risk Management

The motivation for the Reg. 2020/271 was the fact that only six Member States have regulated the norms regarding reclaimed water requirements: Cyprus, Greece, Spain, France, Italy, and Portugal. One of the innovations of the regulation involves the introduction of risk management systems to comply with quality requirements. Risk management systems in the use of reclaimed water are included in the Urban Wastewater Directive. The risk associated to wastewater reuse in agriculture has been analyzed repeatedly, a recent review can be found in ref. [82].

The implementation of risk management systems to the operators of the reclamation plants follows the water treatment chain (see Figure 1), and risk management systems are required between points [b] and [e]. As shown in Table 2, there is no radical change in the definition of parameters to be satisfied, as they are close to existing national norms, but the real challenge lies in the implementation of an assurance protocol. This will be probably the critical point for acceptance. As we have seen, consumer acceptance of reclaimed water use increases with trust in water administration.

The European Regulation differentiates between several situations to establish reclaimed water quality requirements and, consequently, the elements of the risk management system: Crop category, quality requirements, monitoring frequencies, and microorganism indicators.

The requirements established in Annex, describe how the risk management systems should be designed and requirements are related to local conditions (see Annex, point 4). Consequently, a regional strategy should support the reclaimed water user in identifying the various elements, including the identification of hazards for the environment and for public health.

The innovation of the EU‐JRC technical guide^[^ ^50^ ^]^ is the development of risk management protocols for safe reclaimed wastewater us in agriculture to create a risk management plan (RMP). There are critical points that has been addressed in the Guidelines, however, the actual aim of requiring the implementation of risk management systems to comply with the EU Regulation is increase consumers’ confidence in agricultural products irrigated with reclaimed water. Previous sanitary crisis such as “cucumber crisis” in the EU in 2011 where E‐Coli presence in cucumbers in Germany result in the intoxication of part of the population.^[^ ^83^ ^]^ Initially the origin was assumed to be in Southeast Spain where reclaimed water was employed in irrigation but finally it was discovered that origin was an organic farm in Germany. Losses in the crisis were estimated to be more than 200 million EUR supported by the European Union and the City of Hamburg found responsible for the wrong imputation.^[^ ^84^ ^]^

### 5.5 Financing and Cost Recovery

Municipal governments supported by regional, national, and supranational institutions (such as the EU and international donors) are those responsible for building and frequently operating WWT plants where wastewater is treated to reach the legal level of discharge. For example, in the EU, the level is Dir. 271/91 or possibly a stricter local norm (in the case of sensitive areas). The infrastructure and operation are financed, and the cost is recovered by water tariffs, whereby levels close to 100% are reached in certain municipalities or states, and some subsidies are supported (i.e., with a low level of cost recovery) in many locations.

The decision to define the standard of discharge is a political one, as certain countries and regions assume that “water should be discharged with similar quality to water abstracted,” thereby assuming that treatment should go beyond the minimum level set by EU Dir. 271/91 or international norms. The application of stricter norms for wastewater discharge beyond Dir. 271/91 has already been applied in certain EU regions (Murcia, Spain) and Israel. As mentioned in the previous section, the revision of Dir. 271/91 toward higher standards that are stricter after 30 years of implementation should be discussed in the policy agenda. Nevertheless, the most frequent assumption is that urban users are obliged to fulfill Dir. 271/91 for wastewater treatment and the tertiary treatment that takes this level and upgrade the quality to fulfill the current water reuse norm (or future EU Reg.) is the responsibility of the user (irrigator, golf course, industry, etc.).

Nevertheless, once this decision is taken, there is a need to accomplish the financing of infrastructure for treatment, storage, and transport and to cover the operational cost for tertiary treatment and additional services.

A critical factor here is the user's capacity to pay the reclamation and additional cost. It can be observed in Table 1 that 6.6 hm^3^ can be allocated near WWTPs at a cost of less than 0.50 € m^−3^ including all costs except that of storage.^[^ ^85^ ^]^ In this study, the authors assumed a reference cost of the basic tertiary treatment of ≈ 0.08 EUR m^−3^ (cost for the existing 1620/2007) that should be increased by ≈ 0.07 EUR m^−3^ when the new regulation (EU Reg. 2020/741) is implemented. Additionally, transport and distribution from the reclamation plant to the irrigated area should be included, which reaches 0.25 EUR m^−3^ at the most favorable location. This gives a cost close to reference value of 0.50 EUR m^−3^ as the threshold that is difficult to believe that explain the high level reuse estimated by EU services in the mentioned report when typical water cost is in the range 0.02 to 0.10 EUR m^−3^ (Berbel, Borrego‐Marin et al. 2019). It is critical that users can assume this cost, and this can be carried out only for high‐value crops in the form of either vegetables or fruit trees as most of commodities (corn, etc.) cannot support a water cost at the aforementioned levels.

## 6 Discussion

The new EU regulation 2020/271 is aimed to increase water reuse and treat to reach several goals simultaneously: resource recovery, water stress reduction, climate change adaptation, market creation, consumer protection, etc. The regulation is very ambitious, and the process has been supported by stakeholder participation and available scientific and technical knowledge. The use of reclaimed water is seen as an opportunity in water‐scarce regions worldwide.^[^ ^86^ ^]^

According to the results of the survey performed in eight EU countries (more details about the survey provided in Supporting Information), it was detected that the perception of main barriers varies depending on the degree of water scarcity and the importance of irrigated agriculture. Regions with water scarcity focus on water cost and possibilities of consumer acceptance, other more water‐abundant regions focus on social and governance issues to foster collaboration between farmers and food chains.

Policy makers should consider the impact of the new EU regulation, and support farmers in the financing of the operation, at least in the initial stages, to strengthen the risk assurance system that makes transparency and social trust possible. A strong involvement of regional or basin authorities is probably the most efficient mechanism to promote water reuse while avoiding consumer resistance.

Most of the water reuse assumed by the EU impact assessment^[^ ^85^ ^]^ is planned for the southern Mediterranean states. The underlying hypothesis is that current water sources can be substituted by reused water, regardless of the inland or coastal location. This assumption is critical as the EU aims to use 80% of reclaimed water in Italy, Spain, and Portugal. This estimation is based on the ability to pay and the total cost of reclaimed water (excluding storage). This number may be optimistic since the continental inland locations (the majority of irrigated areas in Italy and Spain and a large portion in Portugal are in the upper and medium river basin locations) and the owners of water rights are usually charged ≈0.02 € m^−3^ for surface water and 0.10 € m^−3^ for groundwater^[^ ^19^ ^]^ consequently they are reluctant to change their current source for a new resource that costs 10 to 20 times more (excluding storage). This assumption is unrealistic, and it is not feasible to assume that a policy decision could assign arbitrarily expensive water to farmers. Water rights include the location of the source of abstraction and cannot be changed for a more expensive source without compensation.

In closed basins where there is a moratorium on new irrigated areas (e.g., Southern Spain), the real demand for reclaimed water is for “new users” who lack water rights. This situation where existing water right holders are not interested in the interchange of “conventional rights” for “reclaimed water” mainly for the cost increase should be considered when developing reclaimed water projects.^[^ ^87^ ^]^

Finally, the importance of EU regulation goes beyond EU member states as the influence of EU standards and norms determines the adoption of similar institutions elsewhere and catalyzes technological innovation in third countries. The influence of EU normative worldwide is known as the “Brussels effect”^[^ ^88^ ^]^ making the European Union (EU) is the most influential regulator in the world for domains such as food safety, data transparency, mobile phones, financial instruments, carbon emissions or environmental standards as it is our case.^[^ ^89^ ^]^ Consequently, EU Reg 2020/741 should be seen as a cornerstone of future water reclamation industry.

## 7 Concluding Remarks

Regulation 2020/741 sets new rules that have changed the way EU countries use reclaimed water in agriculture. The political decision to create the norm as a Regulation, thereby avoiding the complex system of Directives and national transposition, accelerating the standard and homogeneous application to all EU regions. This is obviously a positive feature of the new regulation but should also imply an adaptation to local conditions (ranging from Mediterranean islands water‐use stakeholders’ network and in the natural conditions where reclaimed water is used. A technical guide by JRC has recently proposed risk management plans protocols that are an innovative characteristic of the EU regulation.

The success of the new legislation depends on its effective enforcement in EU countries and the practical implementation of certain critical issues, such the roles that different stakeholders should play (farmers, utilities, municipalities), and the definition of the control enforcement agency. Better governance at river basin level is also required, including flexible and efficient water‐rights entitlements and a rational system for sharing and distributing roles and costs among the stakeholders (farmers, municipalities, utilities, risk monitoring agency). The successful adoption of wastewater reuse should be always considering the basin global view and the system water balance to avoid negative impact on downstream users including the environmental flow. It may also open new debates regarding water policy, such as the water reuse for non‐agricultural markets and the on‐going revision of the 30‐year‐old Dir. 271/91 on wastewater treatment, so that all water masses benefit from the improved quality of wastewater treatments.

The new regulation will make the design and innovation of reclamation plants for an EU market and beyond both easier and cheaper and grants technology providers with an opportunity. It will also make it possible to regulate and open the use of reclaimed water and to increase resource use and implement water and nutrients of the circular economy.

## Conflict of Interest

The authors declare no conflict of interest.

## Supporting information

Supporting Information
