# Supplementary material for: Challenges for Circular Economy under the EU 2020/741 Wastewater Reuse Regulation
Source: Glob Chall. 2023 May 26;7(7):2200232. doi: 10.1002/gch2.202200232 (PMC10362123; doi:10.1002/gch2.202200232)
Supplement: Supplementary file 1 — Supporting Information [file GCH2-7-2200232-s001.pdf]

Global Challenges
Open Access

Supporting Information

for *Global Challenges.*, DOI 10.1002/gch2.202200232

Challenges for Circular Economy under the EU 2020/741 Wastewater Reuse Regulation

Julio Berbel, Enrique Mesa-Pérez* and Pedro Simón**

Supporting Information

Survey details

The survey was conducted during the SUWANU-Europe Project with the aim of identifying the strengths, weaknesses, opportunities, and threats in the implementation of reclaimed water initiative within the following European regions: Belgium, Bulgaria, France, Germany, Greece, Italy, Portugal, and Spain.

A total of 105 experts were interviewed following different methodologies: workshop with experts, questionnaires, or round tables. Expert sectors involving is summarized below:

**Key actors' sector**	
Farmers	21%
Private Sector	11%
Drinkwater supplier	2%
Wastewater supplier	14%
National administration	5%
Local administration	11%
Research institution	29%
NGOs	8%

More information about this survey, and the project can be found in the project website (https://suwanu-europe.eu/)
